# The History of Patients after Gastric Operations

**Published:** 1904-03-05

**Authors:** 


### THE HISTORY OF PATIENTS AFTER GASTRIC OPERATIONS.

Mr. Mansell Moullin[1] has followed up the history of eighteen of his patients on whom he performed various gastric operations, chiefly for the cure of gastric erosions and ulcers. He asked each the following five questions:—1. Have you any pain after food? 2. If you have, is it severe? 3. Are you often sick? 4. Have you brought up blood since the operation? 5. Is your general health better? In some cases where erosions of the gastric mucosa were found the base of the erosion was ligatured with absolutely satisfactory results; in other similar cases the hæmatemesis and pain returned after a short respite. It appears that the discomfort arising from perigastric adhesion is relieved to a certain extent after gastroenterostomy, but a more radical operation is necessary to free the patient from pain altogether, and avoid recurrence of the symptom. The only satisfactory method of treating these cases is to cut out the seromuscular base of the adhesion and bring the cut edges together carefully by means of sutures. One of Dr. Moullin's cases was an example of hysterical neurosis, and the patient gave a history of hæmatemesis; her trouble, though relieved for several months after a small erosion had been ligatured, recurred. One case of atonic dyspepsia simulating ulcer was unrelieved by a simple exploratory gastrotomy. Another patient exhibiting all the symptoms of chronic ulcer, including profuse hæmatemesis, after a similar operation recovered much of his health and vigour. The reason of recovery in this case, Mr. Moullin thinks, must have been the free division of pyloric fibres which his gastric incision entailed. When chronic ulcers were excised the result was invariably good whether gastroenterostomy had been performed or not. Two cases of annular pyloric stricture were cured by pyloroplasty.

1 Brit. Med. Jour., Feb. 20th, 1904.

